# MPS1 is involved in the HPV16-E7-mediated centrosomes amplification

**DOI:** 10.1186/s13008-021-00074-9

**Published:** 2021-11-04

**Authors:** Yair Alfaro-Mora, Guadalupe Domínguez-Gómez, Rodrigo E. Cáceres-Gutiérrez, Laura Tolentino-García, Luis A. Herrera, Clementina Castro-Hernández, Rosa María Bermúdez-Cruz, José Díaz-Chávez

**Affiliations:** 1 Departamento de Genética y Biología Molecular, Centro de Investigación y de Estudios Avanzados (CINVESTAV-IPN), Mexico City, Mexico; 2 Unidad de Investigación Biomédica en Cáncer, Instituto de Investigaciones Biomédicas, UNAM/Instituto Nacional de Cancerología (INCan), Mexico City, Mexico; 3 Instituto Nacional de Medicina Genómica, Mexico City, Mexico

**Keywords:** Centrosome, MPS1, PLK4, HPV16-E7

## Abstract

**Background:**

It has been reported that the oncoprotein E7 from human papillomavirus type 16 (HPV16-E7) can induce the excessive synthesis of centrosomes through the increase in the expression of PLK4, which is a transcriptional target of E2F1. On the other hand, it has been reported that increasing MPS1 protein stability can also generate an excessive synthesis of centrosomes. In this work, we analyzed the possible role of MPS1 in the amplification of centrosomes mediated by HPV16-E7.

**Results:**

Employing qRT-PCR, Western Blot, and Immunofluorescence techniques, we found that E7 induces an increase in the MPS1 transcript and protein levels in the U2OS cell line, as well as protein stabilization. Besides, we observed that inhibiting the expression of MPS1 in E7 protein-expressing cells leads to a significant reduction in the number of centrosomes.

**Conclusions:**

These results indicate that the presence of the MPS1 protein is necessary for E7 protein to increase the number of centrosomes, and possible implications are discussed.

**Supplementary Information:**

The online version contains supplementary material available at 10.1186/s13008-021-00074-9.

## Background

The centrosome is a cytoplasmic organelle composed of a pair of orthogonally aligned cylindrical structures. Each cylindrical structure is formed by nine triplets of microtubules organized symmetrically in radial form and surrounded by a pericentriolar protein matrix (PCM), which is responsible for the nucleation of microtubules [1, 2].

Centrioles normally duplicate only once during a cell cycle, and this ensures the presence of two centrosomes and the assembly of the bipolar mitotic spindle for the correct segregation of sister chromatids [3–5]. The presence of multiple centrosomes can form multipolar mitoses, which generate lags in the migration of chromosomes, chromosomal instability, and aneuploidies [4, 6–8]. In addition, abnormal centrosome duplication is one of several defects that can be found in different solid and hematological types of cancer [9–11].

Different mechanisms can generate multiple centrosomes: excessive synthesis of the pre-existing centrioles during a single cycle of cellular replication [12]; de novo formation of centrosomes; or their accumulation when the cells cannot complete cytokinesis and initiate a new round of centrosome synthesis [13]. Several studies have shown that the alteration of the function and/or stability of some proteins like CDK2-cyclin A/E complex [13–15], nucleophosmin chaperone (NPM1) [14], centrosomal protein CP110 [15], phosphatase CDC25B [16] and the kinases PLK4 [17] and MPS1 [18] promote an excessive synthesis of pre-existing centrioles.

MPS1 is a dual kinase that participates in different cellular processes, such as the recruitment of components of the spindle assembly checkpoint (SAC) [19] or duplication of the centrosome [20]. Even though its participation in centrosome duplication has been controversial [21], different reports have described a role by MPS1 in centrosome duplication [20, 22–25]. Its function in the centrosome is regulated by the CDK2/cyclin A complex, which phosphorylates MPS1 in the T468 residue [18]. This phosphorylation prevents the degradation of MPS1 via proteasome by the proteins Ornitin Antizima (OAZ) [26] and Cdkn3 [27], increasing the amount of MPS1 present in centrosome. On the other hand, MPS1 promotes duplication of the centrosome by binding and phosphorylating the Mortalin protein (mtHSP70) at residues T62 and S65 [28]. Interestingly, if the degradation of MPS1 in the centrosome is inhibited [18, 26, 27], an excessive synthesis of centrioles is observed.

Moreover, Duensing and colleagues have reported that the expression of oncoprotein E7 of human papillomavirus type 16 excessively increases the synthesis of centrioles in mononuclear cells, which can promote the maturing of multiple functional centrosomes [29–31]. This HPV16-E7-mediated amplification of centrosomes is independent of E7 ability to bind and degrade the pRB, p107, and p103 proteins [32, 33] since the transfection of E7 in cells deficient in pRB/p53 or pRB/p107/p130 induces the generation of multiple centrosomes [34]. However, for this phenomenon to happen, the participation of CDK2/cyclin AE complex [35, 36], cyclin A transcription [37], and PLK4 kinase RNA deregulation through E2F1 transcription factor [38] are essential.

In this study, we analyze the possible role of MPS1 in the amplification of centrosomes mediated by HPV16-E7. We found that HPV16-E7 increased the mRNA (p < 0.0001) and protein expression of MPS1 in the U2OS cell line. Also, HPV16-E7 increases MPS1 protein stabilization. In addition, we observed that 11.3% (p < 0.01) of cells transfected with HPV16-E7 possessed more than two centrosomes. In contrast, by inhibiting the expression of MPS1 with short hairpin ribonucleic acid (shRNA) or the selective small-molecule inhibitor MPS1-IN-3, the number of centrosomes present in cells transfected with HPV16-E7 decreases at the level of control cells (p < 0.01). These results suggest that the presence of MPS1 protein is necessary to generate an increase in the number of centrosomes mediated by HPV16-E7.

## Results

### HPV16-E7 increases MPS1 transcript and protein levels

**Fig. 1:**
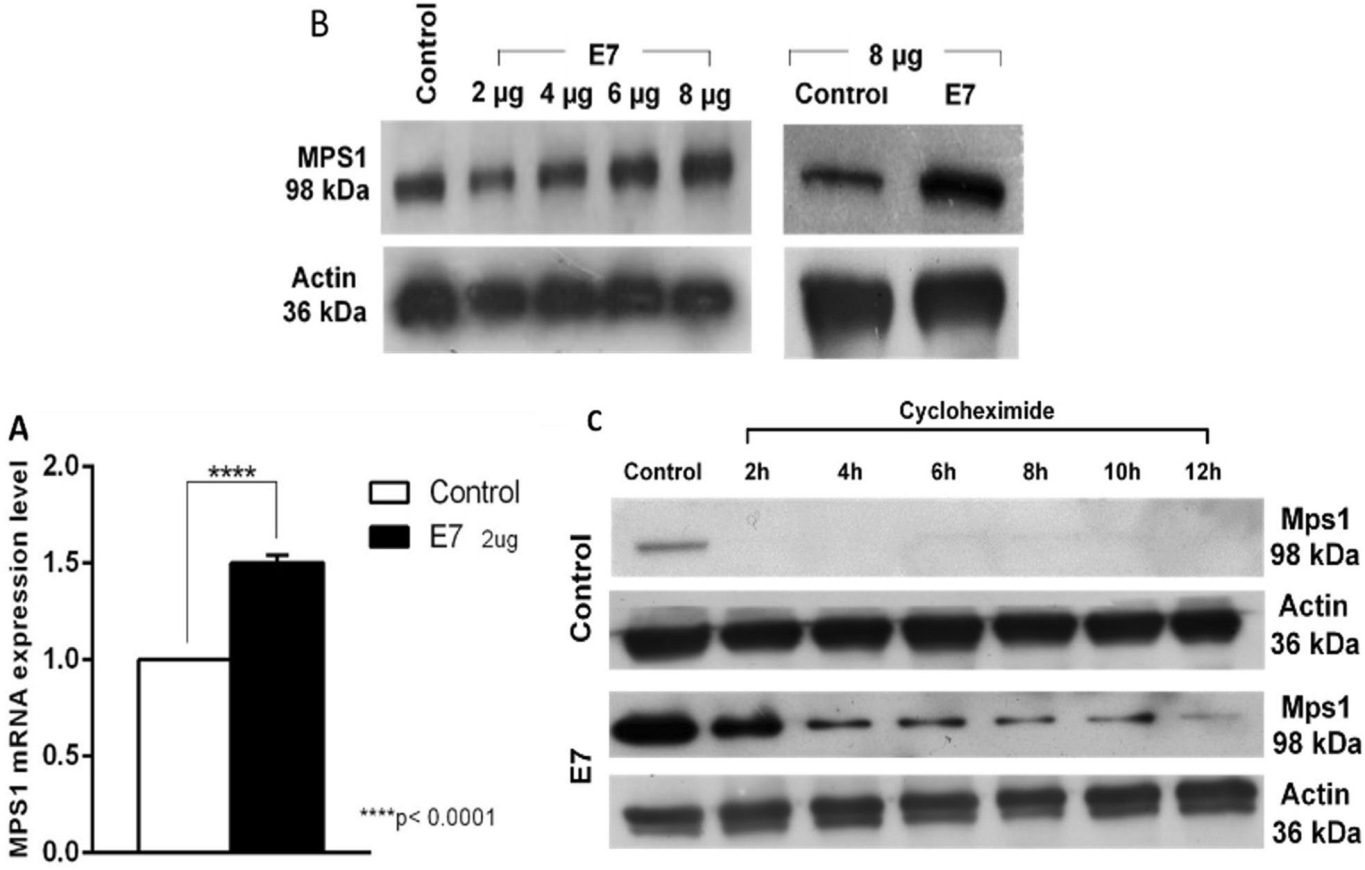
HPV16-E7 increases MPS1 transcript and protein levels. **A** MPS1 expression. MPS1 mRNA in U2OS-E7 cells (2 µg) was determined by qRT-PCR and analyzed in three independent experiments, a statistically significant increase (p < 0.0001) with respect to cells transfected with the empty vector (control) was observed. **B** Western Blot of MPS1. Protein levels were determined in U2OS control cells and U2OS cells transfected with increasing HPV16-E7 plasmid concentrations (2–8 µg, left panel). An increase in MPS1 protein levels was observed in U2OS-E7 (8 µg) with respect to control cells (right panel). Actin was used as a loading control. **C** Stabilization of MPS1 mediated by HPV16-E7. The half-life of MPS1 was determined by treating U2OS-E7 and control cells with cycloheximide at a final concentration of 100 µg/ml. MPS1 protein was more stable in U2OS-E7 cells than control cells

Because E7 deregulates proteins involved in the centrosome duplication cycle, such as PLK4 [35–38], MPS1 transcript and protein levels were determined due it is involved in centrosome duplication [18, 26–28]. An E7 expressing plasmid was transfected in U2OS cells (U2OS-E7, 2 µg), and as a control, we used cells transfected with the empty vector (2 µg), verifying that E7 was expressed only in U2OS-E7 cells (Additional file 1: Figure S1). We analyzed the expression of MPS1 by qRT-PCR and observed a statistically significant increase (p < 0.0001) in the MPS1 expression in U2OS-E7 compared to control (Fig. 1A). Later, we analyzed whether E7 increased the MPS1 protein levels. MPS1 protein levels were analyzed by western blot assay in U2OS cells transfected with increasing concentrations of E7 expressing plasmid (2–8 μg). As a control, we used the highest concentration of empty vector (8 μg), and our results reveal that MPS1 protein levels were increased when transfecting the highest concentration of E7 expressing plasmid as compared with cells transfected with empty vector (Fig. 1B). These results demonstrate that HPV16-E7 induces an increase in both MPS1 mRNA and protein levels. Due we observed an MPS1 protein level increase; we sought if HPV16-E7 could stabilize MPS1 protein. We perform a cycloheximide assay to assess the half-life of MPS1. We treated control and U2OS-E7 cells with 100 µg/mL cycloheximide for 2 h intervals during 12 h. Interestingly, we found that endogenous MPS1 half-life in U2OS-E7 cells was more prolonged until 12 h post-treatment in comparison to control cells where endogenous MPS1 is reduced to 2 h post-treatment (Fig. 1C). These results demonstrate that HPV16-E7 expression in U2OS cells stabilizes MPS1 protein kinase.

### HPV16-E7 mediates centrosome amplification

**Fig. 2:**
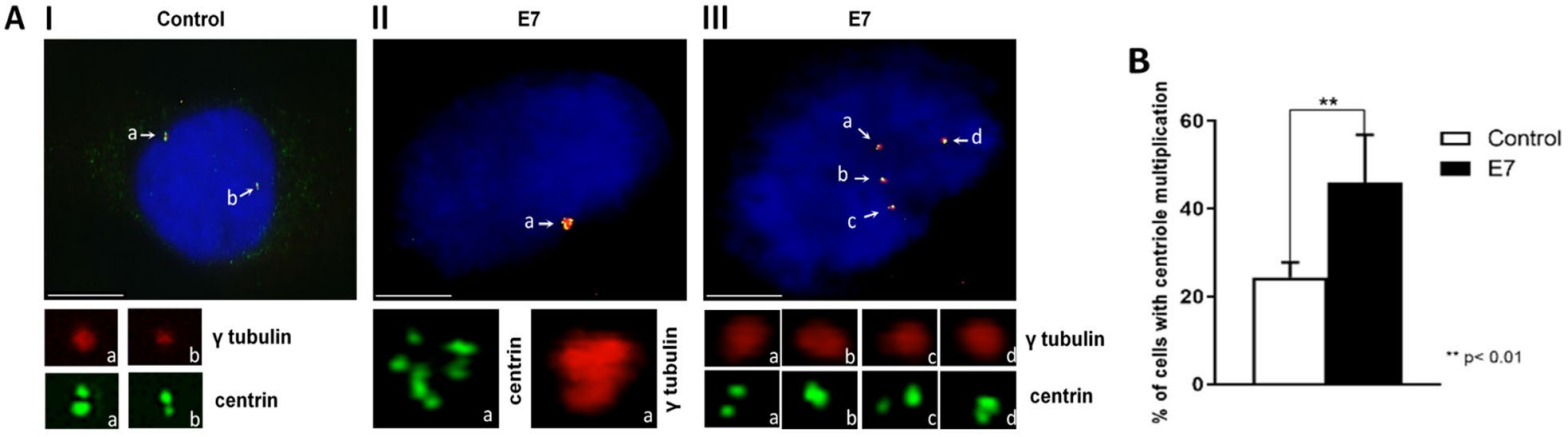
HPV16-E7 increases the centrosome number in U2OS-E7 cells. **A** Immunodetection of centrosomes in cells transfected with HPV16-E7. Immunofluorescence was performed to detect γ-tubulin (red) and centrin (green), contrasting the nucleus with DAPI (blue). We found cells with a γ-tubulin cloud surrounding multiple centrin signals (II) or multiple centrosomes with γ-tubulin signals and two centrin signals (III). The objective used was 100X. **B** Percentage of cells with multiple centrosomes. Mononuclear cells with γ-tubulin and centrin signals were counted in three individual experiments. A statistically significant increase (11.3%, p < 0.01) was observed in cells transfected with HPV16-E7 compared to control cells

Previously, Duensing et al. reported that the amplification of centrosomes was mediated by HPV16-E7 [30]; thus, we decided to assemble their established model to test our hypothesis that MPS1 is involved in centrosome amplification mediated by HPV16-E7. The number of centrosomes present in U2OS-E7 and control cells was determined by immunofluorescence microscopy detecting the presence of two markers: the centrin protein, which indicates the number of centrioles in each centrosome since it is assembled in the distal part of centrioles [39] and the gamma-tubulin protein, which is a centrosome specific marker [40]. For each replicate, 500 mononuclear cells with normal nuclear morphology were identified using centrin and gamma-tubulin signals. We found that U2OS-E7 cells presented centrosome abnormalities like a centrosome with several centrioles (Fig. 2AII) or multiple centrosomes with two centrioles (Fig. 2AIII). While the number of centrosomes with abnormalities present in control cells is 5.2%, 11.3% of U2OS-E7 displayed centrosome abnormalities, a statistically significant increase in the number of cells with multiple centrosomes with respect to control cells was obtained (p < 0.01, Fig. 2B). These results indicate that the E7 expression increases the number of centrosomes in the U2OS cell line, consistent with a previous report [30].

### MPS1 silencing decreases the HPV16-E7-mediated centrosome amplification

**Fig. 3:**
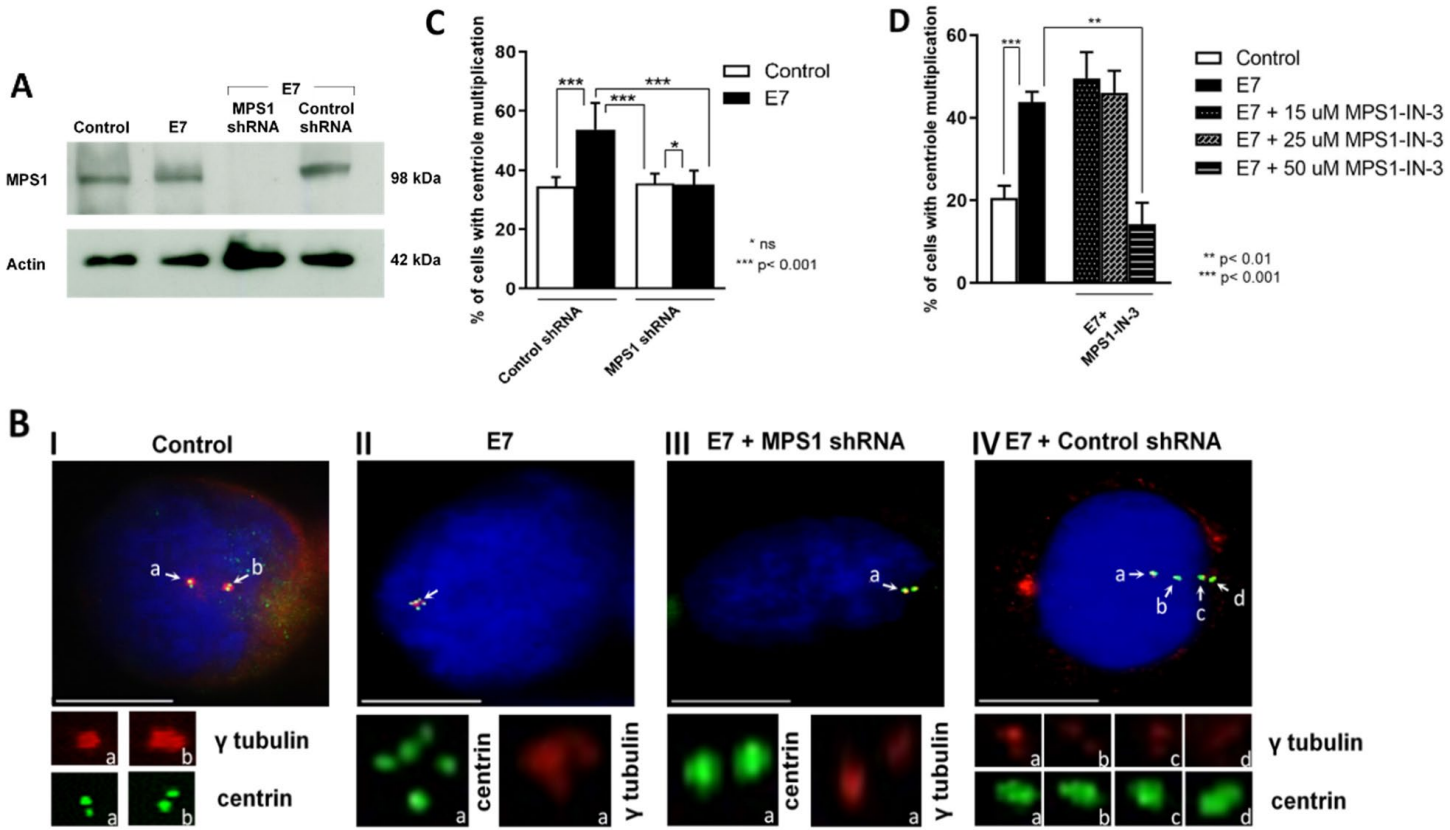
MPS1 silencing or inhibition reduces multiple centrosome presence in U2OS-E7 cells. **A** Silencing of MPS1 by shRNA. MPS1 protein levels in U2OS-E7 and control cells were determined by western blot assay. Also, U2OS-E7 stable expressing cells were transfected with four different short hairpins ribonucleic acid (shRNA) constructs against MPS1 (MPS1 shRNA), thus silencing the MPS1 gene. As a negative control, U2OS-E7 stable expressing cells were transfected with a random sequence (shRNA control), and no effect on MPS1 expression was observed. **B** Immunodetection of centrosomes in U2OS-E7 cells. Immunofluorescence was performed to detect γ-tubulin (red) and centrin (green), contrasting the nucleus with DAPI (blue). Representative images of mononuclear cells with centrosomal abnormalities (γ-tubulin signals and multiple centrin signals) in U2OS-E7 stable expressing cells (II), mononuclear cells with normal centrosome (two γ-tubulin and centrin signals) in U2OS-E7 stable expressing cells transfected with the four shRNA MPS1 constructs (III), and mononuclear cells with centrosomal abnormalities (multiple γ-tubulin and centrin signals) in U2OS-E7 stable expressing cells transfected with a random shRNA construct (IV) are shown. The objective used was 100×. **C** Quantification of the percentage of cells with multiple centrosomes treated with MPS1 siRNAs. Mononuclear cells with γ-tubulin and centrin signals were counted in three individual experiments. A statistically significant higher percentage (p < 0.01) was observed in U2OS-E7 stable expressing cells when compared to control cells. This amplification of centrosomes is significantly decreased by inhibiting MPS1 with shRNAs (p < 0.01). **D** Quantification of the percentage of cells with multiple centrosomes treated with MPS1-IN-3 inhibitor. Mononuclear cells with γ-tubulin and centrin signals were counted in three individual experiments. A statistically significant higher percentage (p < 0.001) was observed in U2OS-E7 stable expressing cells compared to control cells. This amplification of centrosomes is significantly decreased by inhibiting MPS1 kinase activity only with 50 µg MPS1-IN-3 (p < 0.01)

After establishing the study model, we investigated whether MPS1 had any role in the HPV16-E7-mediated centrosome amplification. For this, we transfected four different short hairpin ribonucleic acid (shRNA) constructs against MPS1 and, as a negative transfection control, a scramble shRNA (a random construct) in U2OS-E7 stable expressing cells as well in control cells. After that, MPS1 levels were determined by western blot assay and found that consistent with Fig. 1B, MPS1 protein levels increased in U2OS-E7 significantly compared to control cells. As expected, MPS1 was not detected after transfection of the four shRNA constructs, while the scramble sequence did not affect MPS1 protein levels (Fig. 3A).

Subsequently, we analyzed the MPS1 silencing effect on the number of centrosomes present in U2OS-E7 and control cells by fluorescence microscopy. Mononuclear cells with regular nuclear morphology that presented a positive signal for both centrin and gamma-tubulin were counted randomly (Fig. 3B). We found a statistically significant increase in the percentage of U2OS-E7 cells with more than two centrosomes with respect to control cells (p < 0.01, Fig. 3C), consistent with Fig. 2B. On the other hand, when MPS1 expression was inhibited, the number of centrosomes present in U2OS-E7 cells decreased to levels like control cells (p < 0.01, Fig. 3C). These results suggest that the presence of the MPS1 protein is necessary to generate an increase in the number of centrosomes mediated by HPV16-E7.

To verify that MPS1 presence is necessary for HPV16-E7-centrosome increase, we decided to use the selective small-molecule inhibitor MPS1-IN-3 [41]. We analyzed the MPS1 inhibitory effect on the number of centrosomes present in U2OS-E7 and control cells by fluorescence microscopy. Again, we found a statistically significant increase in the percentage of U2OS-E7 cells that had multiple centrosomes with respect to control cells (p < 0.001 Fig. 3D). We employed an increased amount of MPS1-IN-3 (15, 25, and 50 µM MPS1-IN-3 for 24 h) in U2OS-E7 cells. We observed a significant decrease in the number of cells that had amplification of centrosomes (p < 0.01) when U2OS-E7 cells were treated with the MPS1-IN-3 higher concentration like levels of U2OS control cells (Fig. 3D). This observation also reinforces the possible role of MPS1 presence in the centrosome amplification mediated by HPV16-E7.

### MPS1 and PLK4 could be regulating each other by phosphorylation

**Fig. 4:**
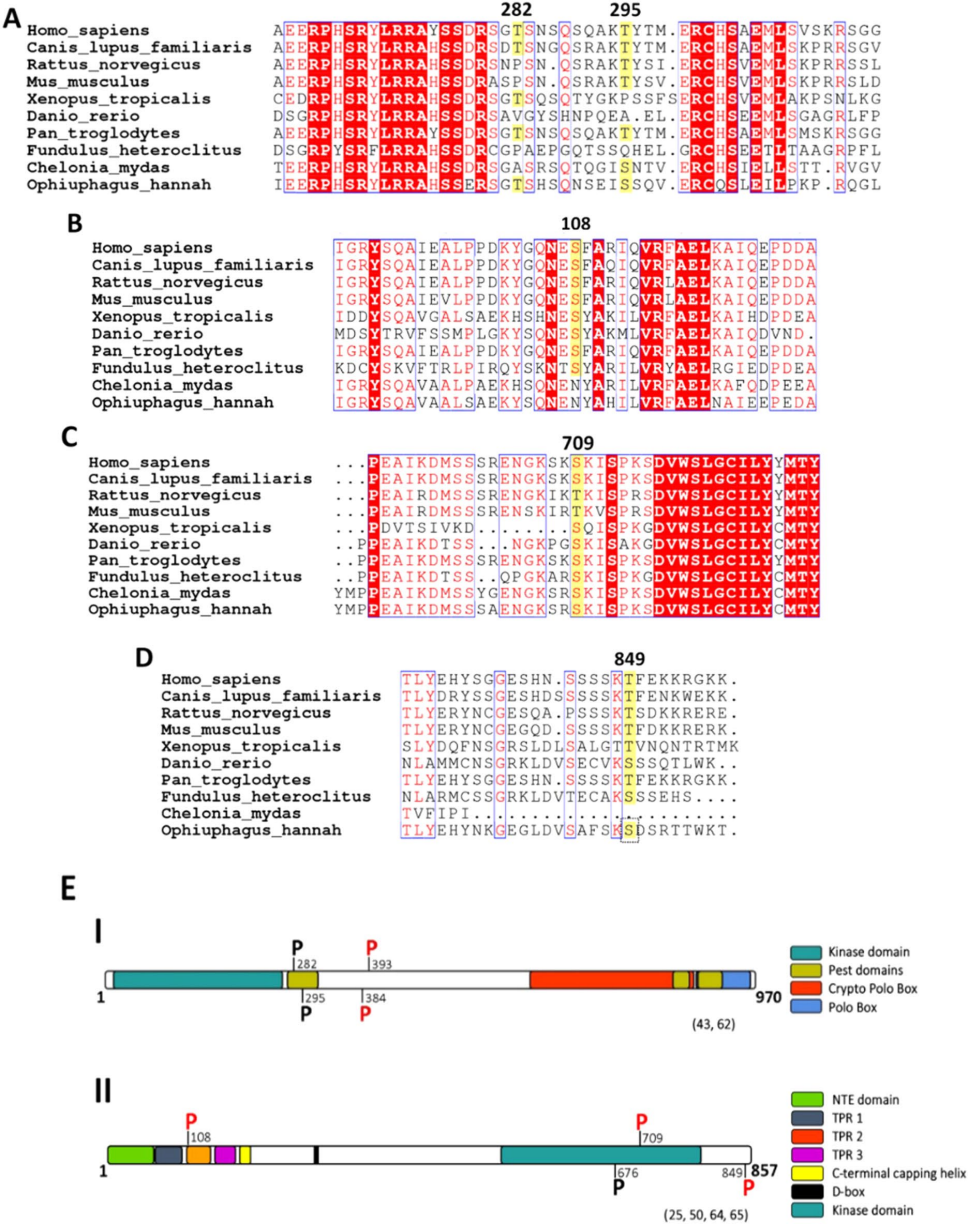
PLK4 and MPS1 protein sequence alignment in humans and other vertebrate species. **A** PLK4 protein sequence alignment in vertebrates. Red letters show homology within a subgroup; the blue boxes show similarity, and red highlights identity. The conserved (blue boxes) and identical (red highlighted white letter) amino acids between species are shown, and residues susceptible to be phosphorylated by MPS1 (yellow) are also indicated. **B**–**D** MPS1 protein sequence alignment in vertebrates. The conserved amino acids between species are shown (blue boxes); identical conserved amino acids are shown (red highlighted white letter), and the residues are susceptible to being phosphorylated by PLK4 (yellow). **E** PLK4 and MPS1 kinase domains. I) PLK4 kinase domains. Four different domains reported earlier, and the possible phosphorylation sites mediated by MPS1 (P red), as well as the reported autophosphorylation sites [45] (P black), are indicated. II) MPS1 kinase domains. Seven domains were previously identified, and the possible phosphorylation sites mediated by PLK4 (P red) as well as the autophosphorylation site [46] (P black) are shown

Previously, Duensing and collaborators identified PLK4 protein as responsible for increasing the number of centrosomes by HPV16-E7 [38]. Based on our results, the consequent question was: What is the mechanism by which MPS1 participates in generating multiple centrosomes by HPV16-E7? It is possible to think that MPS1 and PLK4 could be interacting directly or indirectly to generate multiple centrosomes by the effect of HPV16-E7. To explore this hypothesis, we performed an in silico analysis using the prediction program of kinases-specific phosphorylation sites GPS 3.0 [42] to identify residues in MPS1 and PLK4 susceptible to being phosphorylated. In a complementary way, we used the NetSurfP online program [43], which predicts the secondary protein structure and the accessibility surface of the MPS1 and PLK4 residues likely to be phosphorylated. To validate the data obtained in both programs, we analyzed two residues in PLK4 [44] and one in MPS1 [45], which have already been shown to be phosphorylated in vitro. Table 1 shows the PLK4 residues susceptible to phosphorylation by MPS1 (A) and the MPS1 residues susceptible to be phosphorylated by PLK4 (B), the accessibility of these sites to be phosphorylated, the predictive values of phosphorylation, and the domain where residues are located. This analysis predicts that two residues in PLK4 can be phosphorylated by MPS1, which are conserved in most of the species analyzed (Fig. 4A). The score of these two residues was like that obtained for residues S282 and T295, which have been experimentally demonstrated to be auto phosphorylated in vitro [44]. Our analysis predicts that PLK4 could phosphorylate three MPS1 residues, which are also conserved in most of the analyzed species (Fig. 4B–D). Notably, the S709 residue is present in the MPS1 kinase domain (Table 1B). Similarly, the score obtained from these three residues is like that obtained for residue T676, which has also been experimentally reported to be auto phosphorylated in vitro [45]. These data suggest that both MPS1 and PLK4 could interact directly or indirectly to regulate their activity in generating multiple centrosomes mediated by HPV16-E7.

**Table 1:** In silico analysis of MPS1 and PLK4 phosphorylation sites

A) MPS1	PLK4 aminoacid	Aminoacid in protein	Score	Domain
	T384	Exposed	4.12	
	T393	Exposed	4.25	
Autophosphorylation [43]	S282	Exposed	6.27	Pest 1
Autophosphorylation [43]	T295	Exposed	5.43	Pest 1

B) PLK4	MPS1 aminoacid	Aminoacid in protein	Score	Domain
	S108	Exposed	6.66	
	S709	Exposed	6.66	Kinase
	T849	Exposed	5	
Autophosphorylation [44]	T676	Exposed	5.62	Kinase

**A** Two possible residues exposed in the PLK4 kinase that can be phosphorylated by MPS1 (T384 and T393). **B** Three possible residues exposed in the MPS1 kinase that can be phosphorylated by PLK4 (S108, S709, and T849)

### PLK4 overexpression stabilizes MPS1

**Fig. 5:**
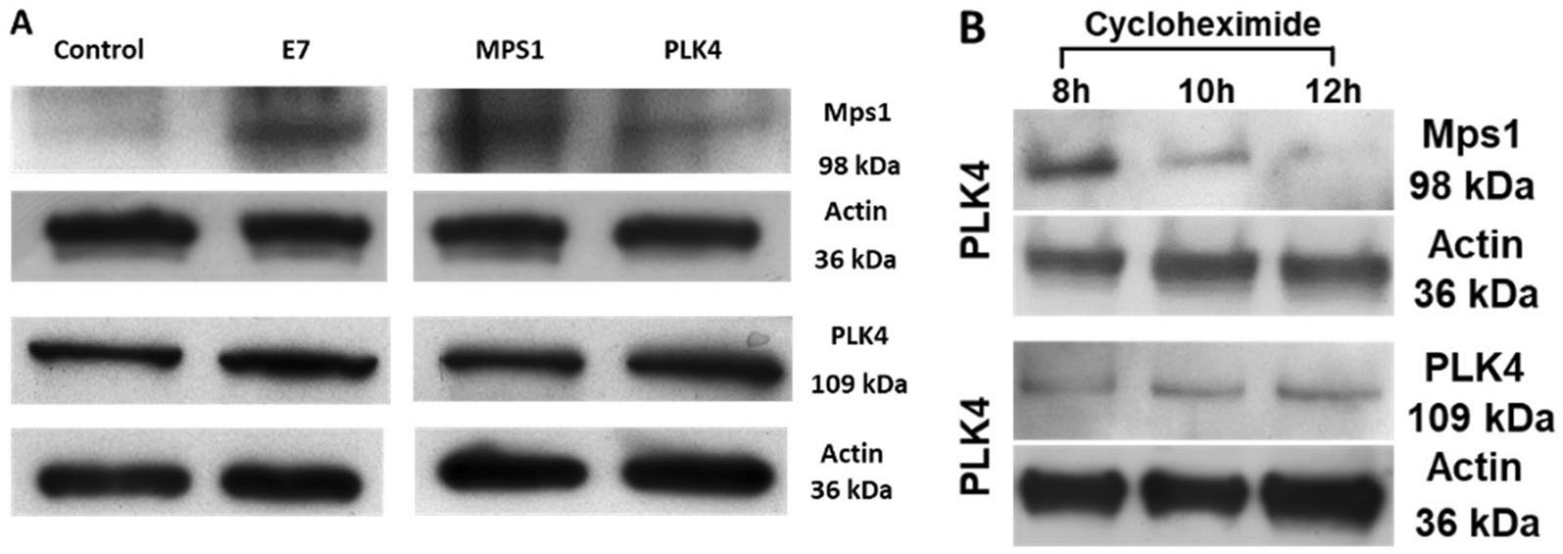
MPS1 stabilization in PLK4 presence. **A** Immunoblotting of MPS1 (row 1) and PLK4 (row 3) in control, U2OS-E7, U2OS-MPS1, and U2OS-PLK4 cell lines (columns). E7 overexpression leads to MPS1 increase, as shown previously (Fig. 1C). PLK4 overexpression increases MPS1 protein levels compared with control cells. Actin was used as load control. **B** MPS1 protein stabilization due to PLK4 overexpression. PLK4 overexpressing U2OS cells shown MPS1 stabilization compared with control cells. Actin was used as load control

To approach our hypothesis that MPS1 and PLK4 could phosphorylate each other to regulate their activity, we generate a cell line that stably overexpresses MPS1 or PLK4, namely U2OS-MPS1 and U2OS-PLK4. We determinate basal levels of MPS1 and PLK4 by Western Blot in the following four cell lines: Control, U2OS-E7, U2OS-MPS1, and U2OS-PLK4. The MPS1 protein levels were increased in E7 expression cells (U2OS-E7) and U2OS-MPS1 compared with control as expected (Fig. 5, row 1). Interestingly, when transfected PLK4 in U2OS cells, we observe that MPS1 protein levels are subtle higher when compared with vector transfected cells (Fig. 5A, row 1) but lesser than E7 and MPS1 over-expressing cells. On the contrary, when we analyzed the PLK4 protein levels on these four cell lines, the PLK4 protein levels were increased in PLK4 and E7 over-expressing cell lines as expected. However, we didn´t see a change in PLK4 protein levels in MPS1 over-expressing cells compared with control (Fig. 5A, row 3). In summary, these results suggest that PLK4 protein kinase overexpression could stabilize MPS1. Otherwise, it seems that MPS1 overexpression does not affect PLK4 protein levels. Therefore, we decided to achieve cycloheximide assay to test this possibility. As we demonstrate in Fig. 1C, we observe a loss of MPS1 protein signal 2 h post-treatment in control cells, whereas, in E7 expressing cells, MPS1 protein is stabilized up to 10 h post-treatment. For that reason, we conducted cycloheximide assay from 8 to 12 h treatment interval in cells overexpressing PLK4 kinase. Interestingly, when U2OS overexpress PLK4 without HPV16-E7 expression, we observed MPS1 protein signal up to 10 h cycloheximide post-treatment (Fig. 5B, row 1) as we observed with HPV16-E7 overexpression (Fig. 1D). Notably, the PLK4 protein levels remain detected up to 10 h cycloheximide post-treatment. Collectively, all these observations suggest that centrosome amplification mediated by HPV16-E7 is driven by an increased HPV16-E7-mediated MPS1 gene expression and maintained by an enlarged MPS1 protein half-life mediated by PLK4. These findings reinforce our in silico analysis showing that PLK4 overexpression increases the MPS1 protein half-life, a kinase involved in the HPV16-E7 mediated centrosome amplification.

## Discussion

The amplification of centrosomes is the main mechanism of generating merotelic junctions and the lag of chromosomes during cell division [7, 8]. These centrosomal defects can be detected in a wide range of tumors in solid tissues [46–48] and various hematological malignancies [49]; in consequence, they are associated with high-degree tumors and a poor prognosis [9]. Several lines of research have demonstrated the ability of oncoprotein E7 from human papillomavirus type 16 in the amplification of centrosomes through altering the centriolar duplication cycle by deregulating several proteins such as CDK2/cyclin E/A, E2F1 and PLK4 [34, 35, 38]. On the other hand, it has also been reported that if the degradation of MPS1 protein is inhibited, the amplification of centrosomes is induced [18, 26]. In the present work, we investigated the possible participation of the MPS1 protein in the amplification of centrosomes mediated by HPV16-E7. MPS1 transcript and protein levels are kept low when the cells enter the phase G1/S of the cell cycle, and they increase in the S phase reaching their maximum point in G2 late/M and decreasing again when the cells re-enter G1 [50, 51]. Several studies have shown that multiple genes regulated during the cell cycle are repressed by the DREAM complex [52–54] and activated by the MMB/FOXM1 complex [54–56] through their union with the cell cycle genes homology region (CHR) present in the promoters of these genes. Both DREAM and MMB/FOXM1 complexes are composed of another protein complex referred as MuvB, which is prepared by the LIN9, LIN37, LIN52, LIN53, and LIN54 proteins. During the phases G0 and G1/S, the MuvB core complex binds with the p130/E2F4-5/DP1-2 proteins forming the DREAM complex, which is present in the promoter of early and late genes of the cell cycle repressing their expression [52, 54, 56]. During the S phase, the DREAM complex dissociates, releasing the MuvB core complex, allowing the B-Myb protein to bind with the MuvB core complex forming the MMB complex. This new complex recruits the transcription factor Forkhead Box Protein M1 (FOXM1), promoting the transcription of cell cycle late genes [54–56].

Interestingly, MPS1 possesses a CHR element at 48 base pairs downstream of its transcription site [57], where the DREAM complex could bind [52]. However, it is not known if the expression of MPS1 is regulated by the DREAM and MMB/FOXM1 complexes, and even if other proteins could be involved, such as the transcription factor E2F1 [58]. In this study, we report that the expression and protein levels of MPS1 increased when using U2OS cells transfected with HPV16-E7 (Fig. 1A and B). We speculate that this increase could be explained if HPV16-E7 dissociation from the DREAM complex is promoted through the degradation of the p130 protein and its interaction with protein complexes composed of E2F1 and DP1-2 [59, 60]. HPV16-E7 could also regulate the expression of MPS1 through the transcription factor B-myb since it has been reported that E7 increases the transcription of B-Myb, generating an increase in the formation of the MMB/FOXM complex. Further, it has been shown that HPV16-E7 can also bind to the MMB/FoxM1 complex generating an increase in the transcription of multiple cell cycle genes [60–62]. These reports are consistent with our observation of the increment in HPV16-E7-mediated MPS1 mRNA and protein levels.

Interestingly, we found that the increase of both MPS1 mRNA and protein levels was associated with the generation of multiple centrosomes mediated by E7. After generating a cell line that stably expresses E7, we observed the amplification of centrosomes (Fig. 2B) consistent with previous reports [30]. Subsequently, using the same model, we diminished the MPS1 expression by shRNAs and blocking the MPS1 kinase activity by the selective inhibitor MPS1-IN-3 (Fig. 3C and D, respectively). This depletion generates a significantly decreased number of cells with multiple centrosomes (Fig. 3C and D). Previously, Tannous et al. demonstrated that employing 2, 5, or 10 µg of MPS1-IN-3 abrogates the spindle checkpoint in U2OS arrested in mitosis where MPS1 has a critical role. However, we did not observe a significant difference in the centrosome amplification employing the doses reported by Tannous et al.; in contrast, a higher MPS1-IN-3 concentration was required to observe the reduced number of cells with multiple centrosomes. This phenomenon could be explained because U2OS cells utilized in this work are stably expressing HPV16-E7, which induces an increase in both MPS1 mRNA and protein levels. Therefore, the inhibitor MPS1-IN-3 concentrations described by Tannous et al. [41] were not sufficient to diminish the overall MPS1 activity and a decrease in the cells harboring multiple centrosomes in our model.

These results correlate with a reduction in the HPV16-E7-mediated generation of multiple centrosomes by inhibiting PLK4 kinase through siRNA, as reported earlier [38], which suggests that MPS1 could be participating in the generation of multiple centrosomes mediated by HPV16-E7 and PLK4. For this reason, we decided to perform an in silico analysis to predict residues susceptible to being phosphorylated by these two proteins. We found phosphorylatable residues in both PLK4 and MPS1, which are conserved in most of the species analyzed (Fig. 4). It has been reported that PLK4 promotes its degradation by autophosphorylating at residues S282 and T295 [44], which are present in the PEST 1 domain [63] (Fig. 4EI). Our in silico analysis showed that residues T384 and T393 in PLK4 are susceptible to being phosphorylated by MPS1 (Table 1, Fig. 4A). These residues are in a non-conserved region between the kinase, PEST1, and cryptic polo box domains (Fig. 4EI). It is currently unknown whether this fragment exerts any control on the protein function in humans. However, Klebba and colleagues reported that, in Drosophila melanogaster cells, the region called Linker 1 (L1) (which is located between the kinase domains, the downstream regulatory element (DRE), and cryptic polo box) exerts an autoinhibitory control over the activation of PLK4 and that this inhibition can be released through the interaction of the cryptic domino box located in the carboxyl-terminal of PLK4 with another protein not yet identified [64]. In this scenario, we hypothesized that MPS1 could induce centrosome amplification by activating PLK4 by its phosphorylation at residues T282 and/or T295. In summary, HPV16-E7 could increase the protein levels of MPS1 and PLK4 and, consequently, generate an increase in the amount of phosphorylated PLK4 protein (presumably by MPS1), resulting in an alteration of the normal centrosome cycle. Although we didn´t find an increase in PLK4 protein levels in the cycloheximide assay, we cannot rule out the possibility that another interaction proposed here can occur that involucres MPS1 activity in the centrosome duplication cycle.

On the other hand, our in silico analysis predicted that the PLK4 kinase could phosphorylate MPS1 in 3 residues: S108, S709, and T849 (Fig. 4B–D). Residue S108 is within the motif TPR2 (Fig. 4EII), which is necessary to determine the location of MPS1 towards the kinetochore along with the motif TPR1 and NTE [25, 65]. It is possible to think that the phosphorylation of MPS1 in residue S108 by PLK4 generates a conformational change in MPS1, which would prevent the dimerization of MPS1 and, therefore, its location in the kinetochore. This phosphorylation could also expose the TPR3 motif and the helix capping [66], which would favor the recruitment of MPS1 towards the centrosome [25]. In 2011, Dhayalan et al. identified 91 different targets of the methyltransferase SET7/9, including the K708 residue of MPS1 [67]. The in-silico analysis detected that PLK4 could phosphorylate the S709 residue of MPS1. These data led us to speculate that the kinase activity of MPS1 could be regulated by a methylation-phosphorylation switch, which would be formed by the phosphorylation of PLK4 in residue S709 of MPS1, preventing methylation of SET7/9 in residue K708 of MPS1 and vice versa [68]. Finally, the MPS1 T849 residue susceptible to being phosphorylated by PLK4 lies outside the kinase domain (Fig. 4DII). It has been found that the mutation of other residues outside the kinase domain can modulate the activity of MPS1 in vitro [23]. Thus, phosphorylation of this residue by PLK4 could increase the activity of the MPS1 kinase and, therefore, increase the number of centrosomes.

Notably, several residues have been identified that are autophosphorylated by MPS1, with one of them being at T849 [23]. However, it has also been described that these sites are substrates for other kinases such as PLK1 [69]; thus, it is possible that PLK4 targets some residues autophosphorylated by MPS1. All these observations can explain the increased MPS1 protein stability observed in our study in U2OS cells overexpressing PLK4. In agreement with this hypothesis, it has been reported that an excessive centriole synthesis occurs when MPS1 degradation is inhibited on the centrosome [18, 26, 27]. It will be interesting to determine if the MPS1 centrosome localization changes when PLK4 is overexpressed or inhibited and how this can alter the centrosome duplication cycle. However, our finding that HPV16-E7-mediated centrosome amplification is due to an MPS1 increased expression of both mRNA and protein levels and MPS1 stabilization mediated by HPV16-E7 and PLK4 suggests a possible cross-regulation between PLK4 and MPS1.

## Conclusions

Taken together, the data shown in this study indicates that MPS1 participates in HPV16-E7-mediated centrosome amplification in U2OS cells.

## Methods

### Cell culture and transfection

The U2OS human osteosarcoma cell line (Cat No HTB-96, Rockville, MD) was acquired from the American Type Culture Collection and grown in McCoy’s 5A modified medium (Gibco, Carlsbad, CA) supplemented with 10% fetal bovine serum (Gibco, Carlsbad, CA) under standard culture conditions.

HPV16-E7 expressing plasmid was donated by Dr. Patricio Gariglio (CINVESTAV, Mexico City). For the transient transfection (48 h) of the U2OS cells, four different concentrations (2, 4, 6, and 8 μg) of plasmid HPV16-E7 and 8 μg of the empty vector (control) were transfected. Transfections were performed using Lipofectamine LTX with PLUS reagent (Life Technologies, Carlsbad, CA) by maintaining the cells in OptiMEM medium (Life Technologies, Grand Island, NY) following the manufacturer's recommendations. For the stable transfection in U2OS, 8 μg of plasmid E7 or 8 μg of the empty vector were used. After 72 h post-transfection, the transfected cells were maintained in culture medium with antibiotic G418 Sulfate (Promega Corp, Woods Hollow Road Madison, WI) at a concentration of 1.2 mg/mL for three weeks. Subsequently, the cells were maintained in culture medium with 100 μg/mL of G418 Sulfate.

The MPS1 silencing was performed in cells stably transfected with HPV16-E7 or empty vector (as negative control). Four shRNA constructs against MPS1 were transiently transfected (OriGene; Rockville, MD) or with a negative (scramble) control (shRNA construct with random sequence) (OriGene; Rockville, MD). Cells were kept in OptiMEM medium (Gibco; Grand Island, NY) and Lipofectamine LTX (Life Technologies, Carlsbad, CA) following the manufacturer's recommendations. The MPS1 inhibition was performed in cells stably transfected with HPV16-E7. The MPS1-IN-3 inhibitor (MilliporeSigma; Darmstadt, Germany) was diluted with DMSO, and three different concentrations were generated: 15 µM, 25 µM, and 50 µM. Cells were grown on slides placed in six-well culture plates at 70% confluence and kept in McCoy's 5A modified medium (Gibco, Carlsbad, CA) supplemented with 10% fetal bovine serum (Gibco, Carlsbad, CA) and the correspondent MPS1-IN-3 inhibitor concentration under standard culture conditions for 24 h. Then cells were analyzed by indirect immunofluorescence.

### Quantitative reverse-transcriptase real-time polymerase chain reaction (qRT-PCR)

Total cellular RNA was extracted using TRIzol reagent (Life Technologies, Carlsbad, CA) following the manufacturer's recommendations. RNA quality was measured using the NanoDrop 2000 spectrophotometer (Thermo Fisher Scientific, Wilmington, DE). 1 μg of total RNA was taken from each sample to synthesize cDNA using the high-capacity cDNA reverse transcription kit (Applied Biosystems, Vilnius, Lithuania) following the manufacturer's recommendations. Real-time PCR was performed with the Step-One Plus Real-Time PCR System (Applied Biosystems, Carlsbad, CA) using 1 μg by the reaction of the reverse transcription product and the SYBR Select Master Mix reagent (Applied Biosystems; Carlsbad, CA) following the manufacturer's recommendations. The RNA sequence of MPS1 (NCBI Reference Sequence: NM_003318.4), 18S (NCBI Reference Sequence: NR_145820.1), and HPV16-E7 (NCBI Reference Sequence: HM211092.1) were obtained from GenBank [70]. The primers used for the amplification of E7 were: (5′-ATG GAG ATA CAC CTA CAT TGC-3′) (forward) and (5′-AAT GGG CTC TGT CCG GTT CT-3′) (reverse). For MPS1 amplification, oligonucleotides (5′-CAG AGG TTC CAG AGA GTA ACC AG-3′) (forward) and (5′- GCT CAA AAG TGG TAT GTT TCT GCT-3′) (reverse) were used. The expression levels of each gene were normalized with the expression of the constitutive gene 18S using the primers (5′-TCG GAA CTG AGG CCA TGA TT-3′) (forward) and (5′-CGA ACC TCC GAC TTT CGT TCT-3′) (reverse). The obtained data were analyzed using the equation 2-ΔΔCT previously described by Livak [71].

### Western blot

Total proteins were extracted using RIPA lysis buffer (50 mM Tris–HCL pH 7.6, 150 mM NaCl, 1% Nonidet P-40, 0.5% sodium deoxycholate, 1 mM EDTA, 1 mM phenylmethylsulfonyl fluoride (PMSF) and inhibitor cocktail of proteases (Cell Signaling Technology; Danvers, MA) for 30 min on ice. The protein concentration was measured using the DC protein assay kit (BioRad, Hercules, CA). Equal amounts of protein were separated through 10% SDS-PAGE and transferred onto a PVDF membrane (Millipore, Darmstadt, Germany). The membrane was blocked for one hour with 5% non-fat milk and incubated with the primary antibody against MPS1 (sc-56968, Santa Cruz Biotechnology 1:500) or PLK4 (sc-100413, Santa Cruz Biotechnology) overnight at 4 °C. Subsequently, striping was performed on the same membrane, blocked with 5% non-fat milk, and the anti-β-actin antibody was incubated (sc-8432, Santa Cruz Biotechnology, 1:500) overnight at 4 °C. The secondary antibody (616520, Invitrogen) coupled to horseradish peroxidase was used at a 1:5000 dilution, and the immunoreactivity was visualized using Pierce ECL Western Blotting Substrate (Thermo Fisher Scientific, Rockford, IL). The densitometric analysis of the immunodetected bands was performed using ImageJ software (National Institutes of Health, Bethesda, Maryland) [72].

### Immunofluorescence

Cells were grown on slides placed in six-well culture plates at 70% confluence. They were fixed with cold methanol for 10 min and permeabilized with cold acetone for 1 min. The non-specific binding was blocked with PBS and 1% albumin for 1 h at room temperature. Primary antibodies anti-centrin 1 (ab11257, Abcam, 1:300) and anti-γ-tubulin (GTU-88, Sigma Aldrich, 1: 1000) were incubated at 4 °C overnight in a humid chamber. Secondary antibodies (ab175700, Abcam, 1:200 and ab150081, Abcam, 1: 500) were incubated for 1 h at room temperature. The DNA was stained with DAPI (Vectashield Mounting Medium with DAPI, Vector Laboratories, Burlingame, CA). The digital images were acquired using Zen lite software (Carl Zeiss, Gottingen, Germany) and AxioImager A2 microscope (Carl Zeiss, Gottingen, Germany) with Axiocam ICc5 camera and an αPlan-FLUAR 100×/1.45 oil objective.

### Quantification of centrosome abnormalities

Each experiment was performed at least in triplicate. Only cells positive for centrin and γ-tubulin were considered, and at least 500 cells of these with normal nuclear morphology in each experimental condition were analyzed. The presence of 2, or 4 positive signals of centrin was considered as a normal number of centrosomes, and the cells with more than four were considered as a centrosomal abnormality.

### In-silico analysis

The sequence of the MPS1 and PLK4 proteins were obtained from the UniProt database [73] and aligned using MUSCLE [74] and ESPript 3.0 [75]. These sequences were subjected to prediction analysis of the secondary structure and protein surface accessibility using the NetSurfP server view. 1.1 [43], also prediction analysis of specific phosphorylation sites of kinases using the GPS 2.0 software was carried out [42].

### Statistical analysis

To measure the differences between mRNA expression means Student´s t-test followed by Welch’s correction was used, and for the number of centrosomes One-Way analysis of variance (ANOVA) followed by Turkey’s test or Brown-Forsythe and Welch ANOVA test were used. These analyzes were performed in the Graph-Pad-Prism program (Version 6.1 for Windows, GraphPad Software, La Jolla California USA, www.graphpad.com). The averages ± the standard deviation were plotted, and a significant difference was considered at a value p < 0.05.

## Supplementary Information

**Additional file 1: Figure S1.** E7 relative expression. E7 expression was calculated in control cells and U2OS-E7 transfected cells. We measure E7 expression values of cervical cancer cell lines reportedly with (SiHa) and without (c-33a) HPV16 integrated genome.

## Data Availability

Not applicable.
