# Supplementary figures and images for: MPS1 is involved in the HPV16-E7-mediated centrosomes amplification
Source: Cell Div. 2021 Nov 4;16:6. doi: 10.1186/s13008-021-00074-9 (PMC8567613; doi:10.1186/s13008-021-00074-9)

# E7 relative expression ( $2^{-\Delta C_t}$ )

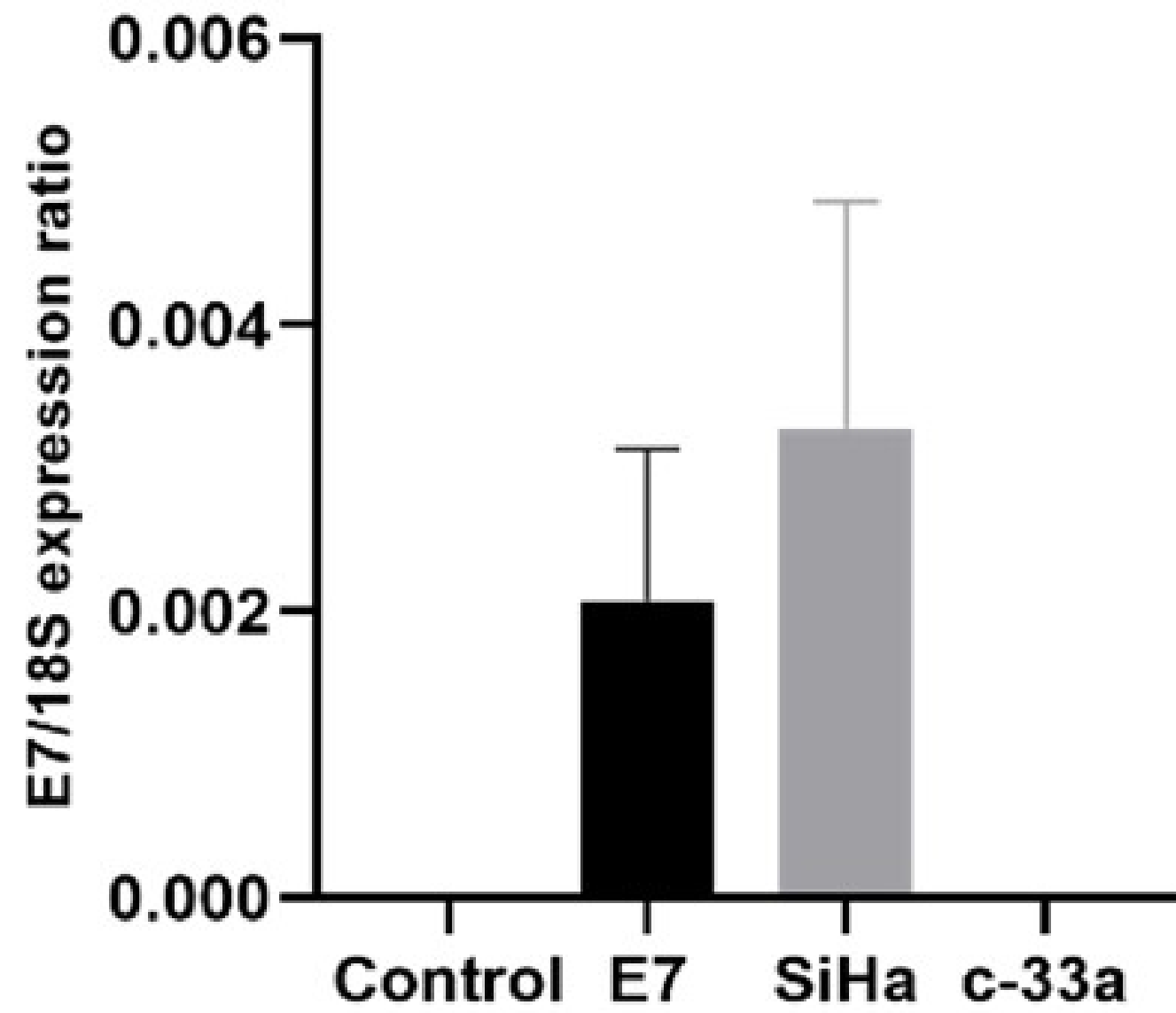

Supplement: Supplementary file 1 — Additional file 1: Figure S1. E7 relative expression. E7 expression was calculated in control cells and U2OS-E7 transfected cells. We measure E7 expression values of cervical cancer cell lines reportedly with (SiHa) and without (c-33a) HPV16 integrated genome. [file 13008_2021_74_MOESM1_ESM.pdf]
